# Fast and intense green emission of Tb^3+^ in borosilicate glass modified by Cu^+^

**DOI:** 10.1038/srep15387

**Published:** 2015-10-21

**Authors:** Fanshu Xia, Siyuan Liu, Yang Wang, Jiayi Mao, Xinxi Li, Yiqun Wang, Guorong Chen

**Affiliations:** 1 East China University of Science and Technology, Key Laboratory for Ultrafine Materials of Ministry of Education, School of Materials Science and Engineering, Shanghai 200237, China

## Abstract

We present photoluminescence properties of Tb^3+^ doped borosilicate glasses modified by Cu^+^. Around 5-time enhanced emission at 541 nm due to the superposed emission of Tb^3+^ and Cu^+^ is observed under the deep UV excitation. Excitation spectra demonstrate a greatly increased absorption of Tb^3+^ ions in the deep UV region towards the Cu^+^ excitation band, while the shortened Cu^+^ emission lifetime of glasses in association with presence of Tb^3+^ ions implies an energy transfer process from Cu^+^ to Tb^3+^ ions. Meanwhile, the Tb^3+^ emission lifetime is significantly shortened from the conventional millisecond level (~4 ms) to the microsecond regime up to around 90 μs. This most likely starts with the role of Cu^+^ as a co-activator by initiating the d-f orbital hybridization process via an interaction with Tb^3+^, thus relaxing the spin forbidden transition of Tb^3+^ ions to the partially allowed one. Moreover, combination of emissions from Cu^+^ and Tb^3+^ ions generates a composite green emission with adjustable CIE (Commission Internationale de L’Eclairage) chromaticity coordinates achievable by co-doping Cu^+^/Tb^3+^ in the different ratio and/or altering the excitation wavelength from deep UV to near UV region.

Rare-earth (RE) ions with the well shielded intra-4f shell transitions give sharp and intense emission lines, and therefore have long been used as activators in different luminescent materials^1^,^2^,^3^. Among RE ions of the most research, Tb^3+^ ions exhibit the transitions occurring dominantly from the excited level ^5^D_4_ down to the lower levels ^7^F_J_ (J = 3, 4, 5, 6) with the emission by the wavelength in the green range^2^,^3^. There have been frequent reports on photoluminescence (PL) properties of Tb^3+^ ions doped in various hosts among which oxide glasses possess high transparency over the ultraviolet (UV) and visible region, high chemical stability and high RE ions solubility so that they have received a lot of interest as perfect host materials to explore the optimum PL properties of Tb^3+^ ions^2^,^3^,^4^,^5^. Majority of investigations in this regard have focused on enhancement of the Tb^3+^ green emission, for example, by the enhanced energy transfer (ET) from co-doped Gd^3+^, Ce^3+^ and Dy^3+^ ions to Tb^3+^ emission centers through a non-radiative energy transfer and a self-sensitizing effect of Tb^3+^ ions^4^,^5^,^6^,^7^,^8^,^9^,^10^,^11^.

On the other hand, Tb^3+^ emissions originating from the forbidden f–f transitions have the long decay time, which is insufficient to satisfy the required fast response time for some applications, e.g., plasma display panels (PDP) and scintillation detectors. Some efforts have been made to develop fast-decaying Tb^3+^ doped green phosphors for 3-D application of PDP^12^. There is also an approach to shorten the emission lifetime of Tb^3+^ activators by preparing the specially core-shell structured LaInO_3_:Tb^3+^@SiO_2_ phosphors^13^. In the present work, the Tb^3+^ doped borosilicate glasses are modified by introducing Cu^+^ as a co-activator with the aim of shortening the Tb^3+^ emission lifetime from the millisecond level to the microsecond regime. The Cu^+^ ion itself also possibly acts as a sensitizer to transfer a portion of the absorbed energy to Tb^3+^ ions, resulting in the expanded and enhanced Tb^3+^ excitations towards the deep UV region. Since great progress of group III-nitride-based UV diodes has been achieved recently^14^, investigations on PL materials to be coupled with deep UV LED chip have become especially potential. Moreover, emissions from Cu^+^ and Tb^3+^ ions cover the blue and green lights, it is thus believed possible to obtain the composite green emission with the adjustable CIE chromaticity coordinates by co-doping Cu^+^/Tb^3+^ in the proper ratio to meet requirements of different applications.

## Results

### Glass samples

Nominal compositions of the glass host for the present work is 20Na_2_O-10BaO-10B_2_O_3_-60SiO_2_ (mol%)^10^. Dopants (Tb, CuO and SnCl_2_) are all extra introduced in the concentration as given in Table 1.

### Emission and excitation spectra and CIE chromaticity diagram

**Figure 1:**
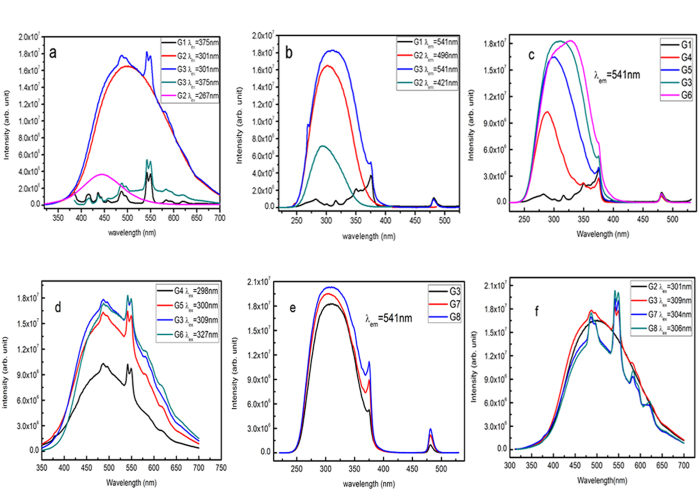
PL (a,d,f) and PLE (b,c,e) of samples G1-G8 (the inset of (a) showing a photo of G3 emission at λ_ex_ = 309 nm).

**Figure 2:**
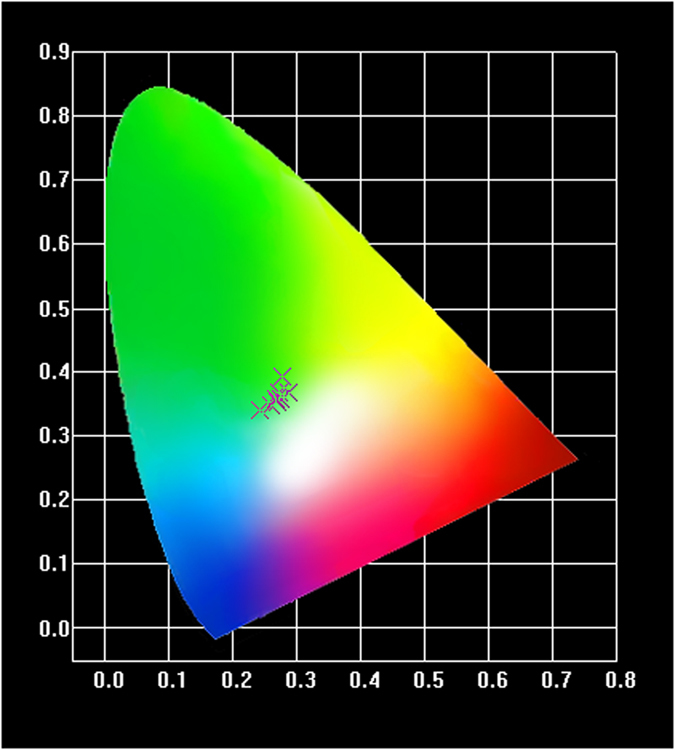
CIE chromaticity diagram of emissions for G1-G8.

**Figure 3:**
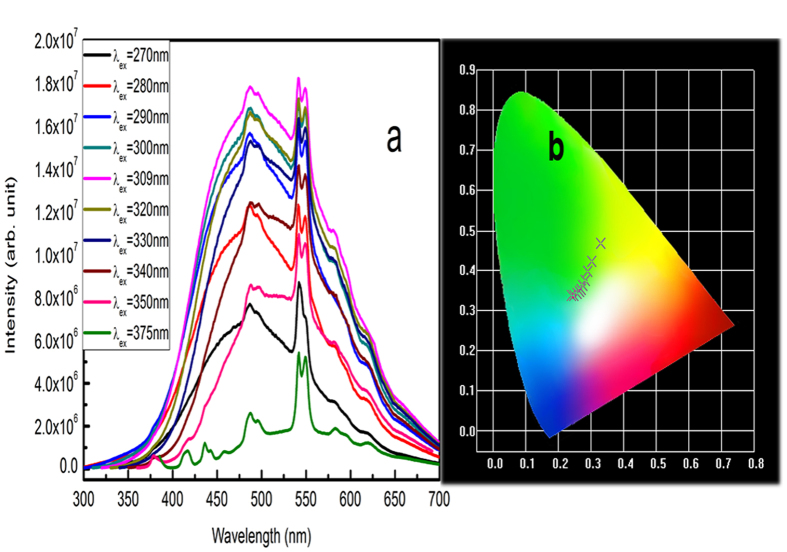
PL spectra of G3 under different excitation wavelength (a) and CIE chromaticity diagram (b).

Photoluminescence (PL) and photoluminescence excitation (PLE) spectra of samples G1–G8 are shown in Fig. 1(a–f), and the corresponding CIE chromaticity diagram/coordinates of emissions are shown in Fig. 2 and Table 2. Figure 3 presents PL spectra of G3 excited by the UV light from 270 nm to 375 nm (a) and the CIE chromaticity diagram indexing an evolution of PL colors (b).

### PL decay curves

**Figure 4:**
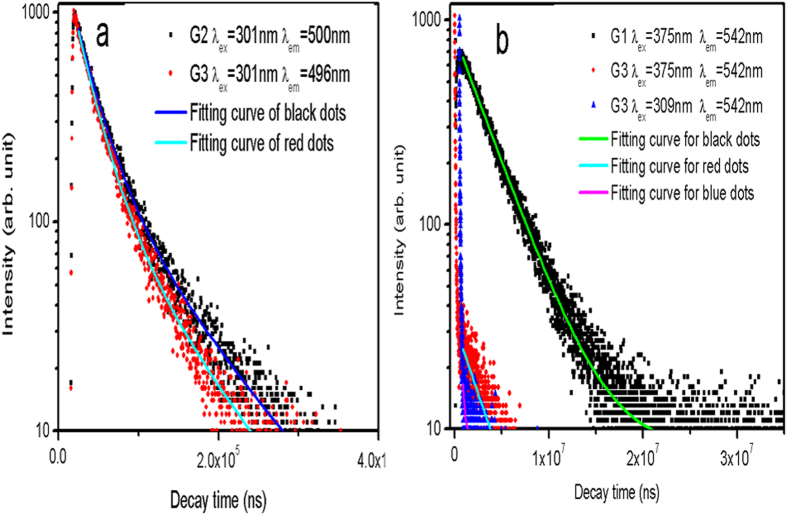
PL decay curves of Cu^+^ (a) and Tb^3+^ (b) emissions for samples G1-G3.

PL decay curves of Cu^+^ and Tb^3+^ recorded on all samples exhibit the strong non-single exponential character from which a proper phenomenological quantification for the emission lifetime of the examined samples was made by the so-called equivalent decay time (τ_eq_) expressed as follows^1^,^10^:


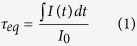


where *I(t)* is the time-dependent emission intensity and *I*_*0*_ is maximum value. As examples, PL decay curves recorded on G1–G3 are presented in Fig. 4, while Table 3 summarizes the calculated*τ*_*eq*_ values of decay curves for all samples.

## Discussion

As shown in Fig. 1(a), the emission lines observed in the spectrum of G1 is under excitation at 375 nm and consist mainly of the violet-blue (410 nm, 430 nm and 453 nm) and green-orange (483 nm, 541 nm, 585 nm and 615 nm) emissions due to electric dipole transitions of Tb^3+^ ions from ^5^D_3_ and ^5^D_4_, respectively, to ^7^F_J_ (J = (6), 5, 4, 3)^7^. By pumping G2 at 301 nm, a broad blue-green emitting band centered at 493 nm is observed and it corresponds to s → d transition of Cu^+^ ions^15^. For G3 when excited at the Tb^3+^ excitation wavelength (375 nm), there appears similar shape of spectrum as for G1 with the increased intensity of emissions in the green-orange region. By exciting at the Cu^+^ excitation wavelength (309 nm), however, G3 shows the composite green emission consisting of Tb^3+^ and Cu^+^ emissions as shown by the inset photo in Fig. 1(a), where the Cu^+^ emission remains the same as that of G2 whereas Tb^3+^ emissions at 483 nm, 541 nm and 585 nm are superposed on the Cu^+^ emission, enhancing maximum around 5 times compared with G1. As G1 and G3 contain the same Tb^3+^ concentration (0.2 mol.%), it strongly suggests that the co-doped Cu^+^ ions act as an effective sensitizer on the Tb^3+^ green emission achievable under the deep UV light excitation.

The predominant role of Cu^+^ ions on the Tb^3+^ emission is further demonstrated by PLE spectra of samples G1-G3 in Fig. 1(b). It is seen that by monitoring the Tb^3+^ emission at 541 nm on G3, beside two PLE bands of G1 at 375 nm and 484 nm (Tb^3+^: ^7^F_6_ →^5^D_3_, ^5^D_4_,), there additionally appears a broad PLE band of G2 ranging from 250 nm to 370 nm (Cu^+^: d → s). Most strikingly, the intensity of PLE bands increases remarkably (maximum by ~10 times) compared with that of G1, approving the beneficial energy harvesting of the Tb^3+^ ions in the whole UV region via Cu^+^ modifying.

According to our previous work, Sn^2+^ ions doped in glasses emit light peaking at around 421 nm under an excitation at 267nm^16^. In order to exclude the influence of Sn^2+^ ions on the Tb^3+^ emission, we also measured PL and PLE spectra of G2 excited/monitored at 267 nm/421 nm, respectively. As compared in Fig. 1(a,b), they are similar to the characteristic Cu^+^ spectra in shape, and more importantly, with an obvious blue shift compared with those excited/monitored at the longer Cu^+^ wavelengths. This phenomenon is identical to the reported results that the continuous shortening of the excitation wavelength of Cu^+^ ions results in a successive blue shift of the respective emission spectra^17^. Thus we believe that the SnCl_2_ is most likely used up for reducing Cu^2+^ into Cu^+^. Therefore, the emission of Sn^2+^ at 421 nm, if any, could be neglected compared with Cu^+^ contributions.

PL decays of Cu^+^ and Tb^3+^ recorded on G1–G3 (Fig. 4) offer the additional evidences supporting the above discussion. Under the excitation at 301 nm on the Cu^+^ singly-doped sample (G2), the τ_eq_ value of the Cu^+^ emission at 500 nm is 39.8 μs (Fig. 4a, Table 3), which is shortened to 34.2 μs up on Tb^3+^ co-doping (G3), consistent with the above suggested ET from Cu^+^ to Tb^3+^. The evaluated energy transfer efficiency *η*_T_ (*η*_T_ = 1 − *τ*_*s*_/*τ*_*s0*_, where *τ*_*s*_ and *τ*_*s0*_are decay times of Cu^+^ with and without Tb^3+^) is ~14.1%, which is too low to account for the drastically enhanced Tb^3+^ emission^18^.

The τ_eq_ value of the Tb^3+^ emission at 541 nm (Fig. 4b) shows that on exciting the Tb^3+^ singly-doped sample (G1) at 375 nm, the τ_eq_ value of the Tb^3+^ emission is obtained typically in the millisecond level (3.79 ms). However, by pumping the sample modified with Cu^+^ (G3) at 309 nm, the τ_eq_ value of the Tb^3+^ emission is substantially shortened to the microsecond regime (0.089 ms). Although Cu^+^ contributes equally to the green emission at 541 nm when excited at 309 nm (Fig. 1a), an excitation at 375 nm can disentangle the effect of Cu^+^ on the Tb^3+^ emission lifetime. In the latter case, the τ_eq_ value of the Tb^3+^ emission is still much shortened to 0.154 ms. We have attributed the relevant mechanism to the role of Cu^+^ as the co-activator in the glass host. Fluorescence of Cu^+^ (d^9^s → d^10^) is known as partially allowed in solids by electronic coupling with lattice vibrations of odd parity, thus being sensitive to host compositions including co-dopants^17^. Accordingly, for Cu^+^/Tb^3+^ co-doped glasses, the d-f orbital hybridization process is most likely initiated as a consequence of an interaction between Cu^+^ and Tb^3+^ ions, thus relaxing the spin forbidden transition of Tb^3+^ ions to the partially allowed one, and finally shortening the τ_eq_ value of the Tb^3+^ emission closer to that of the Cu^+^ emission, as well as achieving the much enhanced Tb^3+^ green emission. Hence, the data of PL, PLE spectra and the τ_eq_ values consistently presents evidences of an enhanced and shortened Tb^3+^ emission in association with the presence of Cu^+^ ions in the present glass host.

As characterized by the CIE chromaticity coordinates/diagram of emissions in Table 2 and Fig. 2, G3 emits green light (0.271, 0.352) under excitation at 309 nm. To tune the PL of the co-doped sample in the green regime, the Cu^+^ or Tb^3+^ concentration is adjusted on the basis of G3 by fixing either Tb^3+^ (G3–G6) or Cu^+^ (G3, G7, G8). In general, increasing the Cu^+^ concentration (G4, G5, G3, G6) enhances and shortens the Tb^3+^ green emission (Fig. 1c, Table 2) as well as expands the Tb^3+^ excitation from the deep UV (298 nm, G4) to the near UV (327 nm, G6) (Fig. 1d). Such a red-shift phenomenon is possibly due to the decreased distances of Cu^+^ which strengthens the Cu^+^-Cu^+^ interaction, thus increasing the ligand field strength surrounding Cu^+^^17^. On the contrary, increasing the Tb^3+^ concentration results in the regularly enhanced excitations without showing obvious shift (Fig. 1e). Moreover, the τ_eq_ value of the Cu^+^ emission at 500 nm reduces little (Table 2), suggesting that the energy transfer efficiency (*η*_T_) from Cu^+^ to Tb^3+^ does not increase further. Instead, the intensity ratio of the Tb^3+^ emission at 541 nm to that at 482 nm increases steadily (Fig. 1f), stemming from the self-sensitizing effect of Tb^3+^ ions with the increased Tb^3+^ concentration^3^,^7^. The CIE chromaticity diagram of emissions for all samples in Fig. 2 and Table 2 show that emissions of Cu^+^/Tb^3+^ co-activators can be tuned effectively in the green regime.

On the other hand, the surrounding crystal field strength for Cu^+^ can be adjusted by changing the excitation wavelength^17^. Therefore, together with the ET from Cu^+^ to Tb^3+^, the successively tunable PL of Cu^+^/Tb^3+^co-doped glasses in the green region becomes possible by altering the excitation wavelength. As shown in Fig. 3, with the excitation wavelength increasing from 270 nm to 375 nm, the intensity of Cu^+^ and Tb^3+^ emissions reaches the maximum at 309 nm and then decreasing (Fig. 3a). Emissions of all samples fall into the green regime as characterized by the CIE chromaticity diagram and can be tuned from the cold moving to the warm and finally to the region close to yellow (Fig. 3b). Thus the tunable green emissions are achieved in G3 by altering the excitation wavelength from the deep UV to the near UV, indicating that Cu^+^, Tb^3+^ co-doped borosilicate glasses are promising as converting phosphors for UV LED chips to generate the required green illumination.

## Conclusions

The fast and intense Tb^3+^ green emission is achieved in Tb^3+^ doped borosilicate glasses modified by Cu^+^. PLE spectra and PL decay curves demonstrate consistently that the enhanced Tb^3+^ emission is in associated with an energy transfer from Cu^+^ to Tb^3+^ ions. The significantly shortened lifetime of the Tb^3+^ emission from ~4 ms to ~0.1 ms implies the possible d-f orbital hybridization process as a consequence of the interaction between Tb^3+^ and Cu^+^, thus relaxing the spin forbidden transition of Tb^3+^ to the partially allowed one. Moreover, the Tb^3+^ emission can be continuously tuned from the cold to the warm in the green regime by adjusting Cu^+^/Tb^3+^ ratio and/or altering the excitation wavelength from deep UV to near UV region. The present work suggests significant potential in PL materials for different applications such as W-LED, solid state display, etc.

## Methods

Glasses for the present work include Tb^3+^ and Cu^+^ singly (G1, G2) and co-doped (G3–G8) samples in the borosilicate glass host. Glass samples were prepared using the melt-quenching method with chemical purity compounds of SiO_2_, H_3_BO_3_, Na_2_CO_3_, BaCO_3_, CuO and elemental Tb as starting materials and SnCl_2_ as the reducing agent (SnCl_2_:CuO as 4:1). Glass batches were prepared according to the nominal compositions as shown in Table 1. The prepared batches were poured into Al_2_O_3_ crucibles and moved to an electric furnace for melting in air at 1550 °C for 3–4 hrs. The melts were quenched in air, and all as-prepared glasses were then annealed at 550 °C for 2–3 hrs, and finally cut into rectangular shapes with 2 mm in thickness and polished to mirror smoothness ready for optical measurements.

All glass samples are transparent in UV and visible region as characterized by transmission spectra (a double-beam Lambda 950 UV-VIS Spectrometer, Perkin-Elmer, USA). There are no obvious absorptions of Cu nanoparticles or Cu^2+^ ranging from 510 nm to 800 nm^15^, indicating that SnCl_2_ as a reducing agent is effective enough to make copper ions exist in Cu^+^. The PL and PLE spectra were collected by a high-resolution spectrofluorometer (Fluorolog-3, Horiba Jobin Yvon Inc., Edison, NJ) using a 450 W Xe-lamp as the excitation source. The PL decay lifetime was measured by FLSP920 (Edinburgh Instruments, Livingston, UK) using nF900 ms pulsed Xe-lamp as source with the pulsed width of 2–3 ms. All measurements were carried out at the room temperature.

## Additional Information

**How to cite this article**: Xia, F. *et al*. Fast and intense green emission of Tb^3+^ in borosilicate glass modified by Cu^+^. *Sci. Rep*. **5**, 15387; doi: 10.1038/srep15387 (2015).

## Figures and Tables

**Table 1:** Extra CuO, Tb, SnCl_2_ doping concentrations (mol.%) and CIE chromaticity coordinates of as-prepared samples.

Samples	G1	G2	G3	G4	G5	G6	G7	G8
Tb	0.2	0.0	0.2	0.2	0.2	0.2	0.6	0.8
CuO	0.0	0.5	0.5	0.1	0.3	0.7	0.5	0.5
SnCl_2_	0.0	2.0	2.0	0.4	1.2	2.8	2.0	2.0

**Table 2:** CIE chromaticity coordinates of emissions for samples G1-G8.

Sample codes	CIE chromaticity coordinates	
	λ_ex_(nm)	Coordinates
G1	375	(0.278, 0.394)
G2	301	(0.274, 0.356)
G3	309	(0.271, 0.352)
G4	298	(0.244, 0.340)
G5	300	(0.278, 0.394)
G6	327	(0.274, 0.356)
G7	304	(0.271, 0.352)
G8	298	(0.244, 0.340)

**Table 3:** Equivalent decay times (τ_eq_) of the Tb^3+^ (λ_e_ = 541 nm) and Cu^+^ (λ_em_ = 496–500 nm) emissions for samples G1-G8.

Samples	Cu^+^/Tb^3+^ (mol%)	Equivalent decay times		
		λ_exc_ (nm)	λ_em_ (nm)	τ_eq_ (ms)
G1	0.0/0.2	375	541	3.790
G2	0.5/0.0	301	496	0.040
G3	0.5/0.2	375	541	0.154
		309	541	0.089
		309	500	0.034
G4	0.1/0.2	298	541	0.098
		375	541	0.731
G5	0.3/0.2	300	541	0.089
		375	541	0.254
G6	0.7/0.2	327	541	0.089
		375	541	0.127
G7	0.5/0.6	304	500	0.034
G8	0.5/0.8	306	500	0.034
